# A deep learning approach to remove contrast from contrast‐enhanced CT for proton dose calculation

**DOI:** 10.1002/acm2.14266

**Published:** 2024-01-25

**Authors:** Xu Wang, Yao Hao, Ye Duan, Deshan Yang

**Affiliations:** ^1^ Department of Electrical Engineering and Computer Science University of Missouri Columbia Missouri USA; ^2^ Department of Radiation Oncology Washington University in St. Louis St. Louis Missouri USA; ^3^ Department of Radiation Oncology Duke University Durham North Carolina USA

**Keywords:** contrast‐enhanced computed tomography, deep learning, medical image processing, proton dose calculation, radiation therapy

## Abstract

**Purpose:**

Non‐Contrast Enhanced CT (NCECT) is normally required for proton dose calculation while Contrast Enhanced CT (CECT) is often scanned for tumor and organ delineation. Possible tissue motion between these two CTs raises dosimetry uncertainties, especially for moving tumors in the thorax and abdomen. Here we report a deep‐learning approach to generate NCECT directly from CECT. This method could be useful to avoid the NCECT scan, reduce CT simulation time and imaging dose, and decrease the uncertainties caused by tissue motion between otherwise two different CT scans.

**Methods:**

A deep network was developed to convert CECT to NCECT. The network receives a 3D image from CECT images as input and generates a corresponding contrast‐removed NCECT image patch. Abdominal CECT and NCECT image pairs of 20 patients were deformably registered and 8000 image patch pairs extracted from the registered image pairs were utilized to train and test the model. CTs of clinical proton patients and their treatment plans were employed to evaluate the dosimetric impact of using the generated NCECT for proton dose calculation.

**Results:**

Our approach achieved a Cosine Similarity score of 0.988 and an MSE value of 0.002. A quantitative comparison of clinical proton dose plans computed on the CECT and the generated NCECT for five proton patients revealed significant dose differences at the distal of beam paths. V100% of PTV and GTV changed by 3.5% and 5.5%, respectively. The mean HU difference for all five patients between the generated and the scanned NCECTs was ∼4.72, whereas the difference between CECT and the scanned NCECT was ∼64.52, indicating a ∼93% reduction in mean HU difference.

**Conclusions:**

A deep learning approach was developed to generate NCECTs from CECTs. This approach could be useful for the proton dose calculation to reduce uncertainties caused by tissue motion between CECT and NCECT.

## 1 INTRODUCTION

Proton therapy is a therapeutic technique that employs high‐energy proton beams to deliver targeted radiation doses to malignant tumors while minimizing damage to neighboring organs and healthy tissues. Accurate dose calculation is crucial for proton therapy. Conventionally, various imaging modalities are utilized to obtain information regarding target delineation and proton range. For example, Non‐Contrast Enhanced CT (NCECT) is commonly used for proton dose calculation, while Contrast Enhanced CT (CECT) is employed to identify tumors and the surrounding tissues. Nevertheless, dosimetry uncertainties arise due to changes in the patient's anatomy and positioning between the NCECT and CECT.

Several studies have explored the impact of CT contrast agents in CECTs on proton range shift. Hwang et al.^1^ studied 20 lung cancer patients and found that the use of contrast agents could cause stopping power overestimation, resulting in additional doses to normal tissues. They suggest using the soft tissue average HU to override cardiac structures in dose calculation. The study by Ates et al.^2^ on 10 pediatric patients with abdominal tumors found that using a dual‐layer spectral detector CT approach could significantly reduce contrast‐related dosimetric error in treatment planning compared to the conventional helical CT approach. Lalonde et al.^3^ compared the influence of contrast agents on proton therapy dose calculation using various CT methods and found that both virtual non‐contrast and electron density and effective atomic number formalisms were less sensitive to the presence of contrast agents for stopping power prediction in phantom studies. However, using dual‐energy techniques or other advanced CT systems can lead to additional patient imaging dose, motion sensitivity, and degraded proton dose calculation accuracy. These systems are also not available for many clinics.

Recent studies have shown Generative Adversarial Networks (GANs)^4^, ^5^ can be very effective for medical imaging modality conversion. Jin et al.^6^ applied GAN to convert CT to MR for radiotherapy treatment, while Brou Boni et al.^7^ and Emami et al.^8^ used GAN to generate CT from MR. Liang et al.^9^ employed GAN to produce CT from cone‐beam computed tomography (CBCT) images.

In this study, we propose a GAN‐based approach to generate NCECT images from CECT images. Our approach only requires CECT images, from which NCECT images are generated by removing the contrast agent. Since NCECT images are synthetically generated from CECT images, there is no tissue motion between these two types of CTs. To the best of our knowledge, this is the first study that uses GAN to convert CECT to NCECT for dose calculation purposes.

## 2 MATERIALS AND METHODS

The initial effort of applying GANs in image‐to‐image translation tasks has inspired a broad range of applications.^10^, ^11^, ^12^, ^13^ In this study, we propose a GAN specifically designed to convert CECT scans to NCECT scans. Unlike many image‐to‐image translation techniques that were commonly implemented in 2D, our model was trained on 3D CT volumetric data, thereby minimizing the inconsistency between slices. Our model also reduced noise and exhibited greater robustness in performance.

### 2.1 Network architecture

**FIGURE 1:**
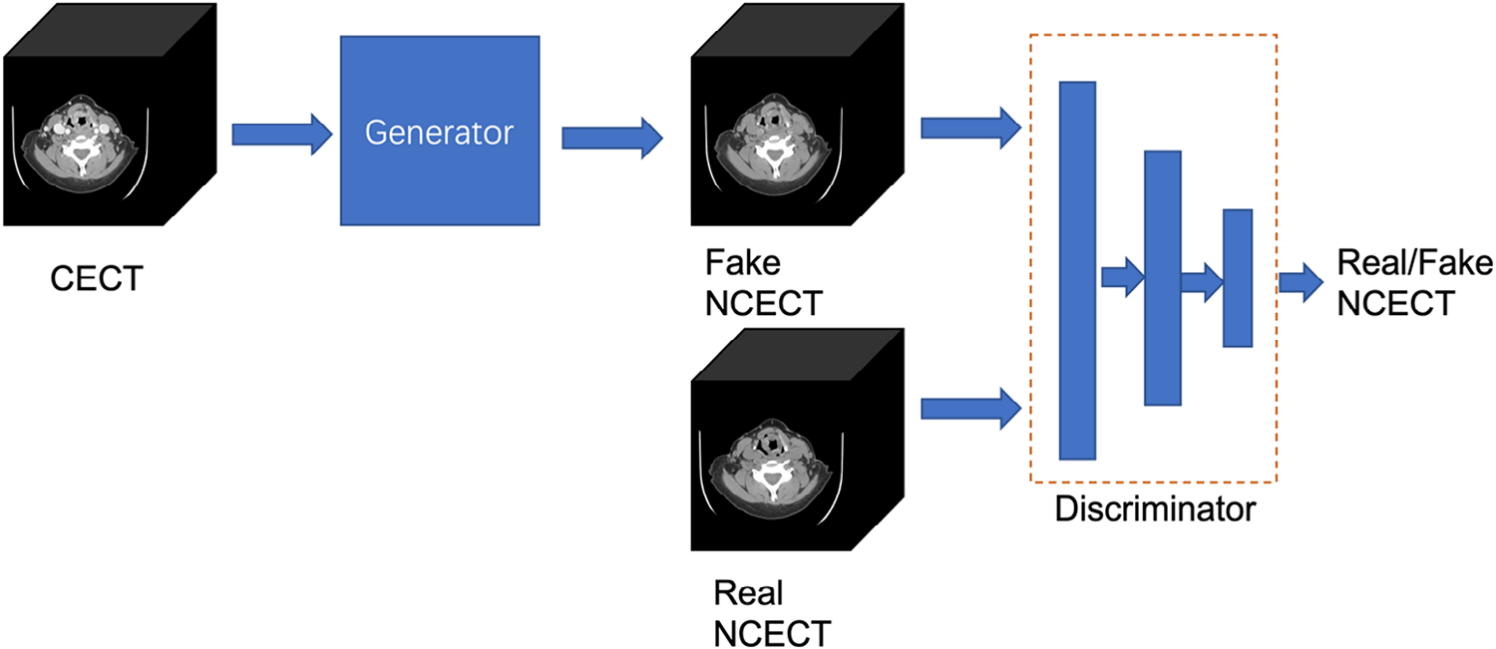
The overall procedure of generating NCECT from CECT. The generator learns to generate the NCECT from a CECT. The discriminator learns to classify between the fake (synthesized by the generator) and real NCECT.

The proposed GAN adapted the design of Pix2Pix,^4^ which included a generator G and a discriminator D. The generator G was responsible for generating NCECT images that were indistinguishable from genuine NCECT scans by the discriminator D. The network architecture is depicted in Figure 1.

#### 2.1.1 Generator and discriminator

Our generator G employed an encoder‐decoder architecture with skip connections to generate artificial NCECT, following the shape of U‐Net. To address the issue of vanishing gradients and expedite training, we substituted the convolution layer in the encoder and decoder with residual blocks. Additionally, we replaced the transposed convolution layer with an interpolation layer to reduce the checkerboard effect in our implementation. The architecture of our discriminator D adopted a patch‐based convolutional neural network (CNN) approach. The final output of the discriminator was a 1D vector of patch‐level scores that determined the likelihood of each patch being real or fake.

#### 2.1.2 Loss functions

In this study, we adopted the use of adversarial losses for our proposed GAN architecture. The losses can be represented by the equation:

(1) $$\begin{array}{c} L_{GAN}\left(G,\;D\right)=E_{x,y}\left[logD\left(x,y\right)\right]+E_{x}[log\left(1-D\left(G\left(x\right)\right)\right] \end{array}$$

where *G* aimed to minimize the objective given input *x* and *D* aimed to maximize it given CECT and NCECT pair (*x*, *y*).

To further improve the quality of the generated image, we also applied L1 loss and perceptual loss (VGG loss). The L1 loss helped to reduce blurring in the generated image, while the perceptual loss measured the difference in features between the generated image and the ground truth. The perceptual loss is expressed as$\sum_{i=1}^{N}\frac{1}{M_{i}}\left|F^{i}\left(x\right)-F^{i}\left(G(x)\right)\right|$. In the equation, $F_{i}$ denotes the i‐th layer with $M_{i}$ elements of the VGG network. The complete objective function for our experiments is as follows:

(2) $$\begin{array}{c} L=\arg\underset{G}{min}\;\max_{D}L_{GAN}\left(G,\;D\right)+\lambda L_{L1}\left(G\right)+\delta L_{vgg}\left(G\right) \end{array}$$

where λ and δ are hyperparameters set to 10 in this study.

### 2.2 Implementation

#### 2.2.1 Dataset preparation and model training

During the training and validation phases, a dataset comprising 20 cases was utilized. These cases were sourced from two publicly available collections, the Clinical Proteomic Tumor Analysis Consortium Pancreatic Ductal Adenocarcinoma Collection (CPTAC‐PDA)^14^ and the Cancer Genome Atlas Stomach Adenocarcinoma Collection (TCGA‐STAD).^15^ Each case consisted of a pair of CECT and NCECT scans, obtained from the treatment plans of the corresponding patient. Among the 20 cases, 16 were randomly selected for training the model, while the remaining 4 cases were employed for validation. The clinical plan results were derived from the data of five new patients.

**FIGURE 2:**
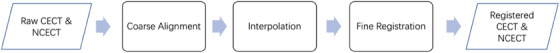
Dataset preparation steps.

To train the CECT conversion model, we registered pairs of CECT and NCECT. The raw CTs consisted of 512 × 512 image slices, with the number of slices ranging from 120 to 200. The three preprocessing steps are shown in Figure 2. During the coarse alignment step, we retained the region of interest and removed any out‐of‐scope slices from the raw CECT and NCECT pair. Next, we interpolated both scans to a dimension of 512 × 512 × 128. A deformable image registration process was utilized at the fine registration step to register the cropped CECT and NCECT. 3D patches of dimension 256 × 256 × 64 were randomly cropped from the registered CT scans to serve as input to the network.

#### 2.2.2 Implementation details

**FIGURE 3:**
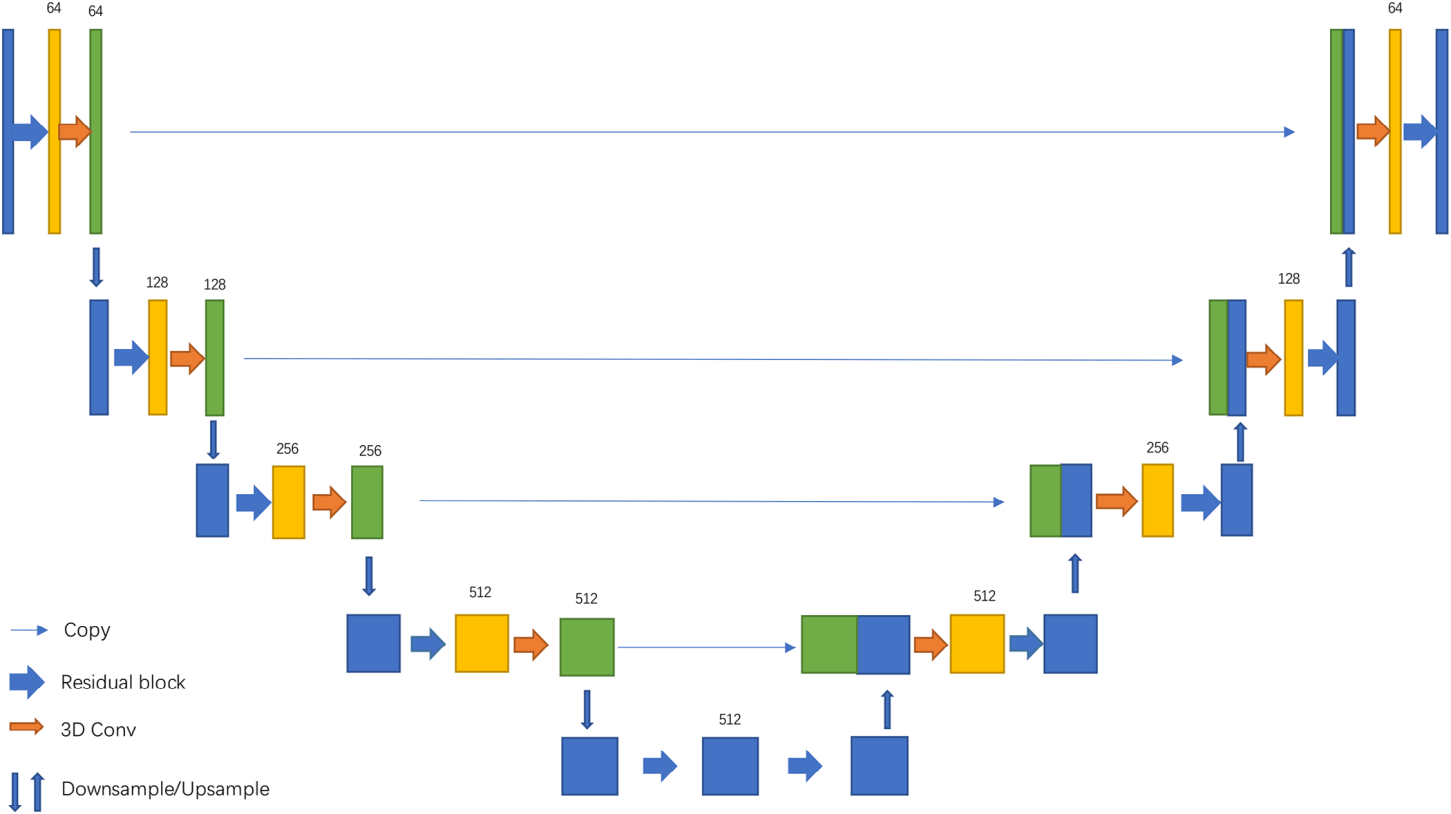
The network architecture of the generator. The generator adopted an encoder‐decoder design with skip connections between mirrored layers in the encoder and decoder. Residual block replaced the convolution layer to thwart the performance degradation problem.

In this study, the Pix2Pix framework was employed, with adaptations made to both the generator and discriminator networks. The generator network consisted of four encoder layers, a residual block serving as the bottleneck layer, and four decoder layers, all implemented in 3D. The encoder layer encoded the 256 × 256 × 64 CT patch to a 16 × 16 × 512 feature space, while the decoder layer projected the feature back to a 256 × 256 × 64 pixel space, generating an artificial NCECT patch. For the discriminator networks, three 3D convolution layers with a stride of 2 were utilized to extract discriminative features. The generator's architecture is illustrated in Figure 3.

#### 2.2.3 GAN model training and inference

The model was trained for a total of 200 epochs. A mini‐batch size of 1 was used. Adam optimizer was utilized to train both the generator and the discriminator. The initial learning rate was set to 0.0002 for the first 100 epochs, and it was linearly decayed over the subsequent 100 epochs. During inference, the generator was executed in the same manner as during training. The CT volume was sequentially divided into overlapping patches of size 256 × 256 × 64. These generated patches were then combined to form the complete volume. To handle the overlapping regions, a simple averaging technique was used.

#### 2.2.4 Clinical proton planning evaluation

In our proton center, both CECT and NCECT images were acquired for proton patients if CT contrast was required for CT simulation, with the NCECT scan acquired first and followed by the CECT scan with IV contrast. The NCECT scan was used as the primary dataset for clinical proton treatment planning. With an Institutional Review Board approval (Washington University School of Medicine. IRB ID # 202305134, approved on 06/05/2023), clinical plans and CT images of five patients, who received proton RT treatments on the MEVION S250i with HYPERSCAN pencil beam scanning technology (Mevion Medical Systems, Littleton, MA, USA), were extracted and used to evaluate the proposed CT contrast removal procedure.

We compared the dose distributions computed from the converted NCECT images against the dose distribution obtained from the reference NCECT images in RayStation 11A (RaySearch Laboratories AB, Stockholm, Sweden). The same method as HWANG et al's^1^ was used to find a range of 90% distal fall‐off. Line dose profiles were drawn within the proton beam path of the field and through the isocenter on transverse images. The proton beam ranges were compared between the CECT and the converted NCECT in the corresponding plans. The proton beam ranges were also measured for the dose plan computed on the true NCECT of the patient. However, there were significant anatomy changes between the true NCECT and CECT for all five abdominal cancer patients. Therefore, the proton ranges computed for the converted NCECT and the true NCECT were not directly comparable.

## 3 RESULTS

### 3.1 Image generation results

**FIGURE 4:**
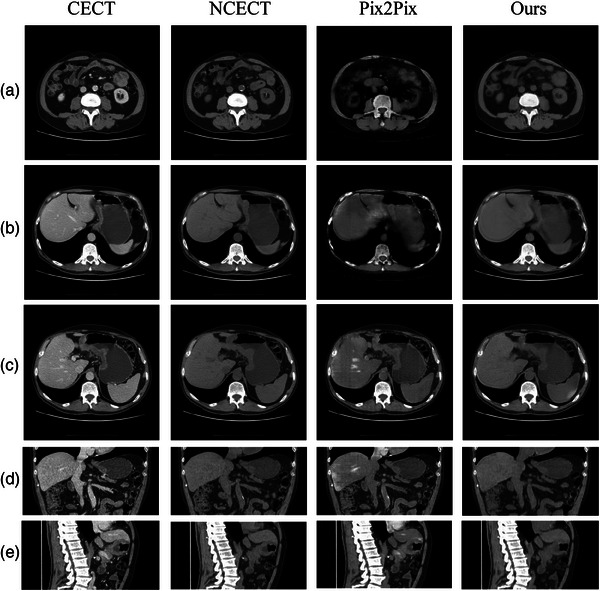
Image generation results from various slices from one of the four validation case. The first column showcases the CECT images. The second column displays the patients’ NCECTs. The third column shows the converted NCECTs using 3D Pix2Pix. The last column shows the converted NCECTs using our method. Our results demonstrate a higher degree of similarity to the real NCECT.

We evaluated the NCECT conversion results on the four cases from the validation datasets. In Figure 4, slices from various views of the CECT of a randomly selected case are presented, along with the corresponding slices of the true NCECT, and the NCECT generated using the regular Pix2Pix method and the proposed GAN method. Compared to the results generated using Pix2Pix, the visual comparison demonstrated that the NCECTs generated using our method exhibit higher similarity to the true NCECTs. Our results demonstrated reduced noise and enhanced preservation of details, indicating the effectiveness of our loss function and optimized network design in generating cleaner NCECT images from CECT. Quantitatively, we used Cosine Similarity and Mean Square Error (MSE) to measure the similarity between the true NCECT and generated NCECT. Cosine Similarity, a measure of similarity between two vectors in an inner product space, is defined as $Similarity\left(Y,\bar{Y}\right)=\frac{Y\cdot \bar{Y}}{∥Y∥∥\bar{Y}∥}$. MSE is calculated as $MSE=\frac{1}{N}\;\sum_{1}^{N}{\left(Y-\bar{Y}\right)}^{2}$, where *N* represents the number of observations, *Y* denotes the real NCECT values, and $\bar{Y}$ represents the generated NCECT values. Table 1 presents the average quantitative results calculated from the four cases. Compared to Pix2Pix, our method achieved higher Cosine Similarity and lower MSE values.

**TABLE 1:** A quantitative comparison between Pix2Pix and our method.

	Cosine similarity	Mean Square Error (MSE)
Pix2Pix	0.499	0.150
Ours	0.988	0.002

The Cosine Similarity and MSE values listed in the table were computed on the true NCECT and the generated NCECT, and then averaged across four cases. Larger similarity and smaller MSE values indicate better performance.

### 3.2 Clinical proton case results

In addition, we conducted a Hounsfield Unit (HU) and mass density comparison using CECT and NCECT images of five proton patient cases, and the generated NCECT images to evaluate the performance of the proposed GAN model. The mean HU difference between the true NCECT and the CECT was 64.52 HU. The HU difference between the true NCECT and the generated NCECT was 4.72 HU. The results, as shown in Table 2, indicated that the generated NCECT significantly reduced the contrast from the CECT.

**TABLE 2:** List of HU and mass density values of selected organs, measured on the true NCECT, the CECT and the generated NCECT for five proton patients.

	Mean/SD (HU)			Mean/SD differences (HU)		Mean/SD mass density difference (%)	
Structures	Generated NCECT	True NCECT	CECT	Two NCECT	True NCECT vs. CECT	Two NCECT	True NCECT vs. CECT
Esophagus	16.7 ± 15.5	14.6 ± 11.1	27.8 ± 12.2	4.8 ± 5.6	13.0 ± 7.3	0.47 ± 0.54	1.26 ± 0.70
Great vessels	38.6 ± 6.7	41.2 ± 6.7	164.1 ± 44.2	5.8 ± 3.3	123.0 ± 43.2	0.45 ± 0.57	6.40 ± 2.58
Heart	21.5 ± 9.3	22.1 ± 3.2	112.4 ± 43.6	5.3 ± 4.3	90.3 ± 41.8	0.51 ± 0.42	5.15 ± 2.46
Left kidney	17.9 ± 13.3	20.1 ± 7.1	83.4 ± 41.8	4.2 ± 6.5	63.3 ± 36.6	0.42 ± 0.64	3.80 ± 1.86
Right kidney	20.9 ± 9.8	21.0 ± 8.6	92.4 ± 44.6	3.5 ± 2.6	71.4 ± 38.6	0.34 ± 0.25	4.28 ± 1.85
Liver	44.7 ± 12.9	46.8 ± 11.8	73.1 ± 23.3	4.7 ± 3.1	26.3 ± 14.6	0.20 ± 0.15	1.15 ± 1.04
**Mean**				**4.7**	**64.9**	**0.40**	**3.67**

The difference values were computed between the two NCECTs and between the true NCECT and CECT.

In Table 2, the mean mass densities corresponding to each structure were interpolated based on the clinical CT curve in the treatment planning system. The mean mass density differences percentage were calculated using true NCECT as reference for both generated NCECT and CECT. The percentages represent the mean mass density absolute deviations from true NCECT. The mean mass density difference between NCECT and CECT is 3.67%, while the difference between the two NCECT scans is 0.40%, indicating that the mean proton stopping power of the generated NCECTs is very close to the true NCECTs.

**FIGURE 5:**
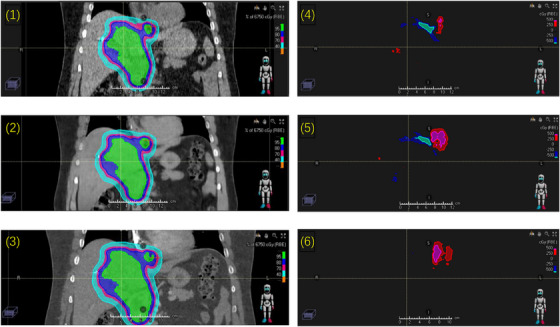
A comparison of the dose plans generated using different CTs and the differences. 1) The dose plan generated using the true NCECT (the planning CT, without contrast). 2) The dose plan generated using the generated NCECT. 3) The dose plan generated using the CECT. 4) The difference between 1 and 2. 5) The difference between 1 and 3. 6) The difference between 2 and 3.

Figure 5 shows the comparisons of the proton dose plans calculated using a patient's true NCECT (the planning CT, without contrast), CECT, and generated NCECT. The dose plans computed using the true NCECT and the generated NCECT exhibited nearly identical dose coverage, except for the superior region, where variations were primarily caused by the different patient's breathing levels. The plan computed using the CECT demonstrated a significant reduction in the dose at the distal end of the beam path. Compared to the dose plan using the generated NCECT, V100% of PTV and GTV changed by 3.5% and 5.5%, respectively in the plan generated using the CECT. The esophagus mean dose changed by 11.4% and the heart D2cc (the dose to maximally exposed 2 cc of the organ) changed by 5.2%. The mean proton beam range shift between the dose plan calculated using the CECT and the generated NCECT was 4.4 mm. The range difference varied between 1.5 mm and 8.2 mm for all the fields. The mean proton beam range shift between the dose plan calculated using the true NCECT and the generated NCECT was 1.1 mm. The range difference varied between 0 and 2 mm for all the fields.

**FIGURE 6:**
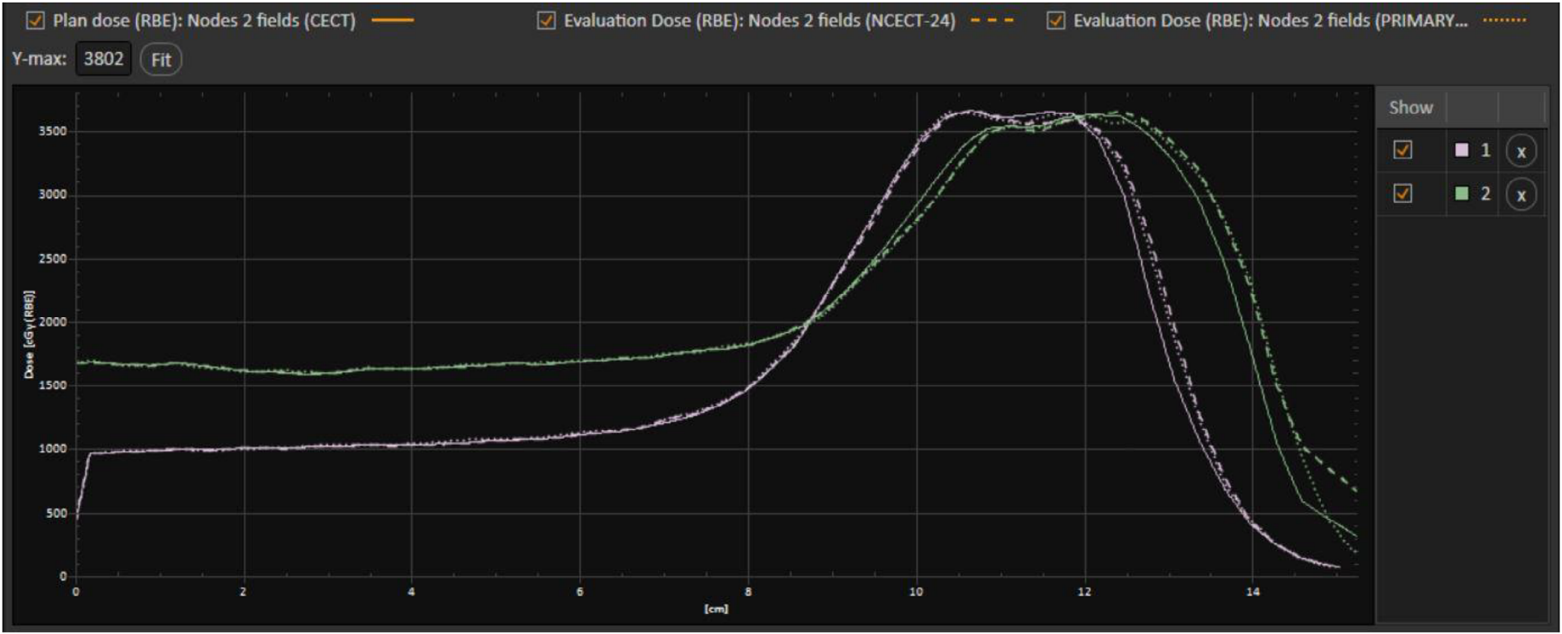
A comparison of the line dose along beam centers of two fields for plans generated using the CECT (solid line), the generated NCECT (long dash line), and the true NCECT, that is, the planning CT without contrast (short dash line).

Figure 6 shows the comparisons of the line dose through beam centers for plans calculated using a patient's true NCECT, the CECT, and the generated NCECT. A clear shift was observed in the CECT plan compared to the two NCECT plans. Small differences existed between the two NCECT plans. These findings suggested that the NCECT generated from the patient’ CECT could be confidently used for proton therapy treatment planning. One should note that the comparison of beam ranges between the clinical plan using the true NCECT to the plan using the generated NCECT and CECT plans was only qualitative. Due to the abdominal organs' motion between the true NCECT and CECT acquisitions, the anatomy in the beam path could be significantly different, for example, more liver tissue with higher HU values versus more fatty tissue with lower HU values.

## 4 DISCUSSION

The results of this study demonstrated the effectiveness of our proposed deep learning approach in reducing contrast from CECTs. The generated NCECT has the potential to improve the efficacy of the clinical proton planning procedure, particularly for tumors in the abdominal area, by eliminating the NCECT scan from CT simulation and reducing the anatomical variations between the contouring CT (the contrast CT) and the dose‐calculation CT (the non‐contrast CT).

Our results agreed with other groups^1^ ^–^ ^3^ that CT contrast could dramatically affect the proton dose calculation. Our solution for CT contrast removal was however very different from previous studies. Compared to Hwang's method of overwriting the density of the segmented organ to the organ's average non‐contrast HU, our method does not require organ segmentation and supports the HU heterogeneity inside and outside the segmented organ, and thus would be theoretically more accurate. Compared to the dual‐energy CT methods by Ates et al. and Lalonde et al., our method only requires the CECT and thus allows simpler CT simulation. Our method does not require the new dual‐energy CT scanners and the dual‐energy scan associated issues, such as limited field of view, poor spectra separation, different motion, and contrast levels between sequential dual‐energy CT acquisitions, and increased imaging dose to the patient.

Although our CECT to NCECT conversion approach yielded promising results, there are still several directions for further improvements. In this study, we utilized a shallow and narrow 3D U‐Net architecture to balance performance and efficiency. However, more advanced and memory‐efficient network architectures are worth exploring.^16^ Furthermore, the size of image patches can affect the generated NCECT's consistency, as overlapping regions may not match up. We addressed this issue in our implementation by increasing the size of overlapping regions and averaging the results. The method can be improved by learning the consistency between neighboring patches. In future work, we plan to include additional training data, to cover the broad range of CT contrast intensity variations, which would be beneficial for improving the model inference performance. We also plan to quantitatively analyze the sensitivity of the proposed CT contrast removal procedure against CT contrast variations, and therefore quantify the model robustness and generalizability.

In the future, this GAN model can be extended to support additional image modalities and body parts. Our model was only trained on abdominal CT data; however, with proper training, it could also support head and neck CT data.

## 5 CONCLUSION

This study presented a novel GAN model for generating NCECT using CECT as the input, and the results indicate that our approach is potentially useful for streamlining the proton dose calculation process.

## AUTHOR CONTRIBUTIONS

*Conception and design*: Deshan Yang and Yao Hao. *Implementation*: Xu Wang and Deshan Yang. *Data acquisition*: Deshan Yang and Yao Hao. *Result analysis*: Xu Wang, Deshan Yang and Yao Hao. *Writng and final approval*: all authors.

## CONFLICT OF INTEREST STATEMENT

There is no conflict of interest for any of the authors.
